# Construction of Macroporous Co_2_SnO_4_ with Hollow Skeletons as Anodes for Lithium-Ion Batteries

**DOI:** 10.3390/gels8050257

**Published:** 2022-04-21

**Authors:** Jintian Wang, Junzhang Wang, Xingzhong Guo, Hui Yang

**Affiliations:** 1 State Key Laboratory of Silicon Materials, School of Materials Science and Engineering, Zhejiang University, Hangzhou 310027, China; 21926060@zju.edu.cn (J.W.); 12026080@zju.edu.cn (J.W.); yanghui@zju.edu.cn (H.Y.); 2 Hangzhou Global Scientific and Technological Innovation Center, Zhejiang University, Hangzhou 311200, China

**Keywords:** Li-ion battery, Co_2_SnO_4_, sol-gel method, hollow skeleton macroporous structure

## Abstract

Increasing the energy density of lithium-ion batteries (LIBs) can broaden their applications in energy storage but remains a formidable challenge. Herein, with polyacrylic acid (PAA) as phase separation agent, macroporous Co_2_SnO_4_ with hollow skeletons was prepared by sol-gel method combined with phase separation. As the anode of LIBs, the macroporous Co_2_SnO_4_ demonstrates high capacity retention (115.5% at 200 mA·g^−1^ after 300 cycles), affording an ultrahigh specific capacity (921.8 mA h·g^−1^ at 1 A·g^−1^). The present contribution provides insight into engineering porous tin-based materials for energy storage.

## 1. Introduction

In recent years, rechargeable batteries as portable energy storage and supply devices have attracted considerable attention. Lithium-ion batteries (LIBs) are a very widely investigated battery technology because of their high energy density and stable cycling performance. Tin-based materials, one of the promising anode materials for high-rate high capacity lithium (Li)-ion batteries (LIBs), offer high theoretical specific capacity of 990 mAh·g^−1^ (4.4Li + Sn → Li_4.4_Sn), processability, high ionic conductivity, and capability of rapid charge and discharge [1]. Moreover, compared with the metal tin (Sn), stannate (M_x_SnO_y_) is believed to be a more promising anode candidate for LIBs due to its smaller volume changes during lithium charge and discharge, and it also exhibits higher ionic conductivity than simple stannic oxide (SnO_2_) [2]. However, the practical application of M_x_SnO_y_ anode is hindered by two main issues: (i) the inherent poor electrical conductivity leads to the low utilization of M_x_SnO_y_, impairing the high-rate capability; and (ii) the unstable solid-electrolyte interphase (SEI), limiting the long-cycling stability.

The most common strategy for enhancing high-rate capability and long-cycling stability is structuring the electrodes appropriately to decrease the diffusion length. A particularly effective way of achieving short path lengths for ion diffusion is to employ porous electrodes. Yuvaraj et al. prepared Co_2_SnO_4_ particles using a sonochemical method, which had a capacity of 777 mAh·g^−1^ after 20 cycles at 40 mA·g^−1^ with a capacity retention of 67% [3]. However, previous studies mainly focused on textural porosity (voids created by the packing of particles), which still could not attain a stable capacity, and few studies use the true porosity (bicontinuous pores and walls).

It has been demonstrated that stannate (M_x_SnO_y_) coupled with hierarchical porous materials are viewed as realistic candidates for LIBs because the porous structure may facilitate electrolyte infiltration, increase utilization of active material, reduce ion diffusion distance, and provide void spaces for variations in volume of active material during cycling, resulting in enhanced stability and conductivity. However, materials with too high porosity will lower the packing density, such as carbon aerogels, so they will be flooded by electrolytes, making the device performance less attractive for practical application. Thus, there is considerable interest in creating materials that have proper porosity for lithium-ion batteries with high energy densities [4].

Herein, we demonstrate the fabrication of a high-performance Co_2_SnO_4_ anode with considerably enhanced cycling stability and rate capability by a sol-gel process accompanied by phase separation. The amount of PAA significantly affected the phase separation process, which resulted in controlled morphologies and properties of the Co_2_SnO_4_ anode. The bicontinuous macroporous Co_2_SnO_4_ anode was synthesized by proper amount of PAA; a too small amount of PAA was undesirable because the incomplete phase separation process weakened the skeleton strength to fragment into particles, while a too large amount of PAA led to excessive phase separation to form a closed-cell structure. Subsequently, crystalline Co_2_SnO_4_ could be yielded by the heat treatment process. This bicontinuous macroporous structure led to an enhanced rate performance of the Co_2_SnO_4_ anode by promoting the transfer of electrons and Li ions. It is worth noting that this structure could be applied to other anode materials of lithium-ion batteries.

## 2. Results and Discussion

### 2.1. Formation Mechanism of Macroporous Co_2_SnO_4_/C Composites

**Figure 1:**
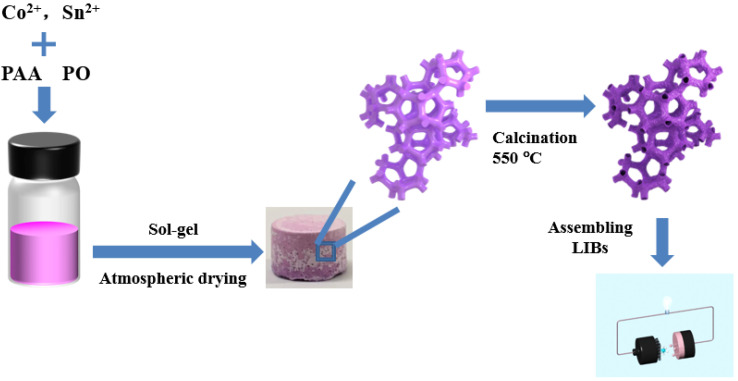
Schematic illustration for the synthesis of hollow skeleton macroporous Co_2_SnO_4_.

Figure 1 shows the preparation process of hollow skeleton macroporous Co_2_SnO_4_. Firstly, cobalt-tin hydroxide gel was prepared by sol-gel method combined with phase separation using cobalt source (CoCl_2_), tin source (SnCl_2_), phase separation agent (PAA), and gel accelerator (PO) as precursors. With the hydrolytic polymerization of Co^2+^ and Sn^2+^ in solution, amplitude modulation decomposition occurred when the composition of the sol-gel system was in the amplitude modulation curve. If the composition was near the critical composition (the maximum value of the free energy composition curve) during the sol-gel transition, the co-continuous structure composed of two interconnected phases was “frozen” in the gel. In this case, one phase was mainly composed of cobalt tin hydroxide and phase separation agent, and the other phase was composed of solvents. Therefore, the co-continuous macroporous cobalt-tin hydroxide xerogel block could be obtained under atmospheric pressure drying condition, and then heat-treated at 550 °C, the composition in the middle of the porous framework diffused outward, and finally a stable hollow skeleton was obtained. When the amount of phase separation agent was too small, the phase separation was not sufficient in the sol-gel process, and the strength of the skeleton structure formed by cobalt-tin hydroxide was too low, which was easy to break into smaller nanoparticles and could not form a continuous skeleton structure. When the amount of phase separation agent was too large, the phase separation was too fast to fully separate the solute phase from the solvent phase, and most of the skeleton structure was closed pore. It can be seen that the phase separation agent is the key to control the formation of porous structure, which can not only make the metal salt solution form gel blocks, but also promote the uniform diffusion of the gel skeleton in the process of heat treatment, thus forming a hollow skeleton. Therefore, only with appropriate amount of phase separation agent can the co-continuous hollow skeleton macroporous structure be formed.

### 2.2. Pore Structure and Microstructure of Macroporous Co_2_SnO_4_/C Composites

**Figure 2:**
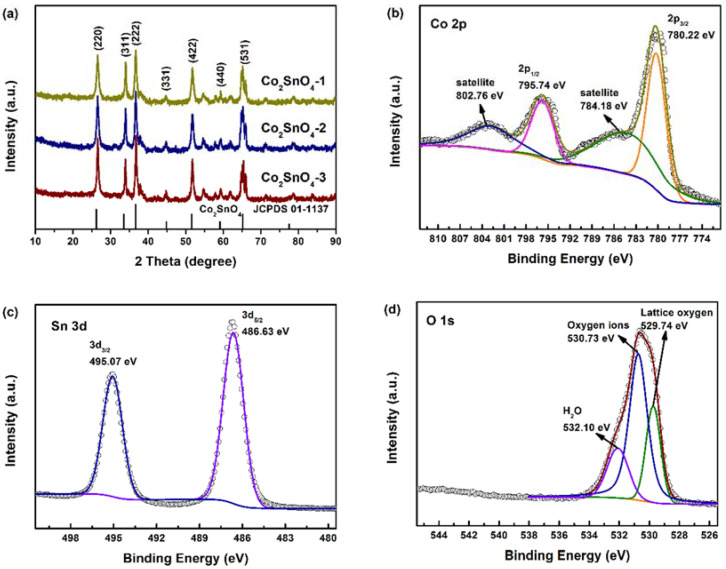
(**a**) XRD patterns of Co_2_SnO_4_-1, Co_2_SnO_4_-2, and Co_2_SnO_4_-3. (**b**–**d**) High-resolution XPS spectra of Co 2p, Sn 3d, and O 1s for Co_2_SnO_4_-2.

XRD patterns of both samples in Figure 2a show dominant peaks at 26.12, 33.54, 36.65, 51.59, and 65.19, corresponding to the cubic Co_2_SnO_4_ phase structure (JCPDS 01-1137). No impurity peaks were detected, revealing that the addition of different amounts of PAA does not affect the phase structure of the product.

The detailed elemental chemistry status of Co_2_SnO_4_ was further investigated with XPS. The survey of Co_2_SnO_4_ (Figure S1) displays the signals of Co, Sn, and O. The Co 2p spectrum shown in Figure 2b possesses several peaks. The peaks at 780.22 eV and 795.74 eV are related to the 2p1/2 and 2p3/2 levels of Co^2+^ in Co_2_SnO_4_ [5], while the Co 2p peaks around 784.18 eV and 802.76 eV are regarded as Co-containing satellite peaks [6]. As for the Sn 3d region in Figure 2c, two peaks of Sn 3d5/3 and 3d3/2 of Sn^4+^ in Co_2_SnO_4_ at 486.63 eV and 495.07 eV were clearly observed [7]. The O 1s (Figure 2d) could be deconvoluted into three species as lattice oxygen, oxygen ions, and H_2_O at 529.74, 530.73, and 532.10 eV, respectively. Among the three kinds of O species, the contents of oxygen ions were more than the other two species. It has been reported that the oxygen ions are beneficial to improving the activity of electrode materials.

The TG-DSC curves of Co_2_Sn(OH)_6_-1, Co_2_Sn(OH)_6_-2, and Co_2_Sn(OH)_6_-3 xerogels were measured in air atmosphere as shown in Figure S2. The mass decline and exothermic peak of dry samples prepared by Co_2_Sn(OH)_6_ with different PAA content were investigated. The TG curve shows that the pyrolysis of the three samples was carried out in three steps: from room temperature to 200 °C, from 200 °C to 350 °C, and from 350 °C to 450 °C. The weight loss below 200 °C came from the evaporation of water physically adsorbed in the skeleton, and the weight loss around 300 °C came from the pyrolysis of organic groups in the gel and the removal of Cl^−^ containing groups (there are a lot of Cl^-^ ions from CoCl_2_ and SnCl_2_ in the gel network, which may coordinate with Co (II) and Sn (II) or play a role of charge compensation in the network). The weight loss around 400 °C came from the gel conversion to Co_2_SnO_4_ [8]. From Co_2_Sn(OH)_6_-1 to Co_2_Sn(OH)_6_-2 and then to Co_2_Sn(OH)_6_-3, with the increase of PPA content in the precursor, the weight loss increased gradually from 200 °C to 350 °C, and at the same time, the weight loss increased from 350 °C to 450 °C [9]. In addition, on the DSC curve, with the increase of PAA content, the temperature point of the sharp exothermic phenomenon in the three samples shifted to high temperature, and the intensity of the exothermic peak increased gradually.

**Figure 3:**
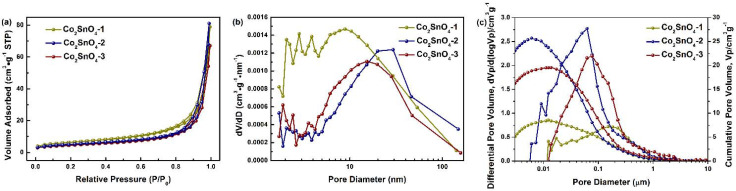
(**a**) Nitrogen adsorption/desorption isotherm, (**b**) pore size distribution plot, and (**c**) macropore size distributions of Co_2_SnO_4_-1, Co_2_SnO_4_-2, and Co_2_SnO_4_-3, respectively.

The pore properties of the obtained samples were evaluated by nitrogen adsorption/desorption isotherm measurements. All samples exhibited typical type IV isotherms with notable type H3 hysteresis (Figure 3a), demonstrating the presence of mesoporosity [10]. The Brunauer–Emmett–Teller (BET) surface areas, pore volume, and pore sizes for the prepared samples were summarized in Table 1. Co_2_SnO_4_-2 displayed higher specific surface area (SSA, 22.31 m^2^·g^−1^) than Co_2_SnO_4_-1 (18.04 m^2^·g^−1^), implying the formation of highly porous structure as the result of phase separation of adequate amount PAA. The SSA was 16.55 m^2^·g^−1^ for the Co_2_SnO_4_-3 sample. In addition, the pore distributions revealed the mesoporous structure (Figure 3b), and the average pore diameter (APD) was larger than 20 nm (Table 1). With the appropriate addition of PAA, the pore size distribution was narrow and the APD increased. The higher SSA and porous structure with appropriate PAA content imply that PAA was conductive to the formation of highly developed porosity, but an excess amount of PAA would block the pore. PAA played a vital role in optimizing the porosity and morphology, which was possibly caused by the stretching of macromolecular chains during phase separation and the release of more volatile molecules from the PAA during the high-temperature pyrolysis. Particularly, the external surface area was predominant in all samples (>85%, Table 1), with Co_2_SnO_4_-2 possessing the highest proportion of 91% and the highest pore volume of 0.13 cm^3^·g^−1^ (Table 1) among the Co_2_SnO_4_-n [10]. Figure 3c shows the large pore size and volume data obtained by mercury porosimetry. It can be seen that when the addition of PAA was 1.43 g, the total pore volume was the largest, the pore size distribution was inclined to the smaller large pores, and the average pore size was about 60 nm. However, when the addition of PAA was 0.71 g, the porous skeleton structure could not be formed due to insufficient phase separation, and most of the pores obtained were stacked pores of nanoparticles, so the volume of macropores was the smallest. When the addition of PAA was 2.14 g, the excessive phase separation process and short phase separation time caused the skeleton pores to be gelated and frozen before they were fully opened, leaving a “developed” closed pore structure. It can be seen that using the right amount of PAA product can make the hole bigger, and the macroporous structure was conducive to improving the electrochemical performance of materials, that is, the well-designed macroporous structure can better alleviate the volume change of tin-based materials through high specific surface area and rich free space [11], and because of its open pore wall structure, it could also accelerate the diffusion of lithium ions [12].

**Figure 4:**
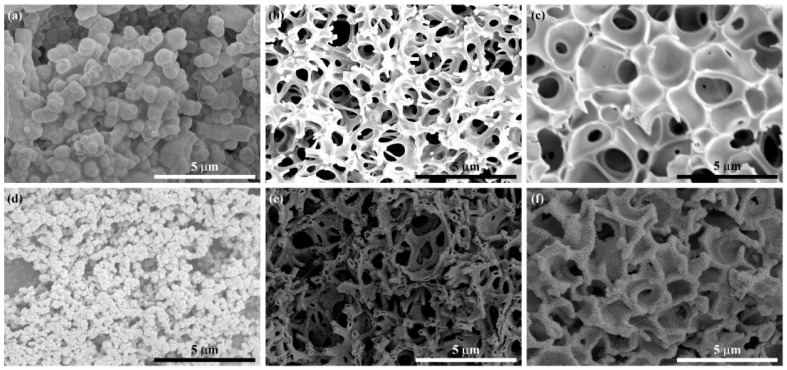
SEM image of (**a**) Co_2_Sn(OH)_6_-1, (**b**) Co_2_Sn(OH)_6_-2, (**c**) Co_2_Sn(OH)_6_-3, (**d**) Co_2_SnO_4_-1, (**e**) Co_2_SnO_4_-2, and (**f**) Co_2_SnO_4_-3.

Figure 4a–f shows the SEM images of cobalt-tin hydroxide xerogels and Co_2_SnO_4_ prepared with three different PAA additions. It was found that the bicontinuous macroporous structure develops with the increase of the amount of PAA in Figure 4a–c. With the increase of m_PAA_ from 0.71 to 2.14 g, the xerogels changed from particle aggregation morphology to bicontinuous morphology, indicating that increasing the amount of PAA could promote the phase separation of the sol system and form a rich macroporous skeleton structure. When the excess m_PAA_ increased to 2.14 g, the pores of the xerogels were closed, which is obviously due to the fact that the interface could drive the gel skeleton to coarsen gradually, and excessive PAA made the tendency of phase separation too strong and the tendency of phase separation too large, which inhibited the formation of pores. Therefore, a good bicontinuous macroporous morphology can be obtained by adding the appropriate amount of PAA. Figure 4d–f shows the SEM images of the three samples after heat treatment. It can be seen that the nanoparticles of Co_2_SnO_4_-1 were smaller than those before heat treatment, the adhesion between the particles became less, and the stacking pores became more. Co_2_SnO_4_-2 maintained the bicontinuous macroporous skeleton structure before heat treatment. Miraculously, the skeleton presented a hollow morphology, which will provide a larger expansion space for the volume change during the cycle as an anode material, but also reduce the ion diffusion length, while Co_2_SnO_4_-3 still maintained a closed porous structure after heat treatment, and the skeleton was relatively thick.

**Figure 5:**
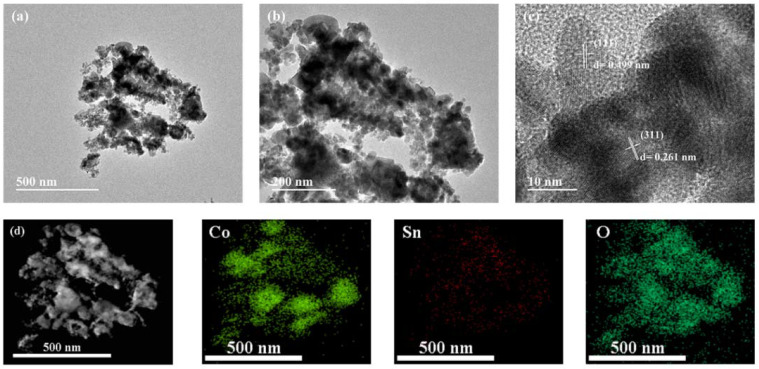
(**a**–**c**)TEM image, and (**d**) the corresponding elemental mapping images of Co, Sn, and O elements, respectively.

The structure of Co_2_SnO_4_-2 was studied by TEM. The image of Figure 5a clearly shows the microstructure of the Co_2_SnO_4_ formed in the range of about 500–600 nm, which is connected by the aggregation of spherical particles to each other. Figure 5b clearly shows the spherical particles vary in size, ranging from 20 nm to 130 nm. Figure 5c depicts the lattice stripes (0.499 and 0.261 nm) of Co_2_SnO_4_ corresponding to the X-ray diffraction peaks of (111) and (311), respectively [13]. In addition, the EDX spectrum (Figure 5d) shows that the main elements were cobalt (Co), tin (Sn), and oxygen (O), without any other impurities, indicating that the prepared material had a high purity [14].

### 2.3. Electrochemical Performances of Macroporous Co_2_SnO_4_/C Composites

**Figure 6:**
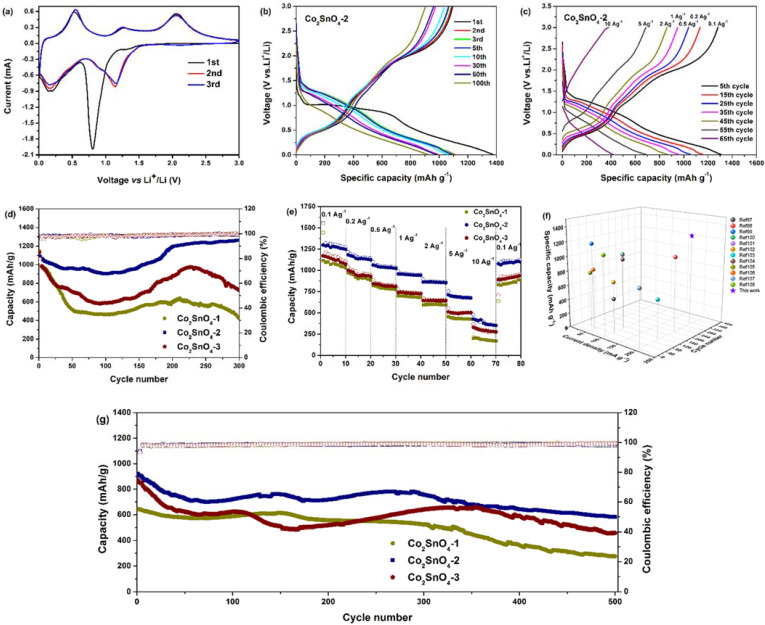
Lithium storage performance of Co_2_SnO_4_-2 anode: (**a**) CV profiles at 0.1 mV·s^−1^, (**b**) the first, second, third, fifth, tenth, thirtieth, fiftieth, and hundredth galvanostatic charge/discharge profiles at 200 mA·g^−1^, (**c**) rate performance. Lithium storage performance of different anodes: (**d**) Cycling stability at 200 mA·g^−1^ and (**e**) rate performance. (**f**) Electrochemical performances comparison between tin-based and cobalt-based oxides anodes with different methods and components. (**g**) Long cycling performance of Co_2_SnO_4_ anodes at 1.0 A·g^−1^ for 500 cycles.

In addition, the electrochemical performance of Co_2_SnO_4_ electrodes was also studied. Figure 6a shows a typical cyclic voltammogram (CV) of Co_2_SnO_4_-2. The electrochemical process of Co_2_SnO_4_ electrode can be attributed to the mechanism of electrochemical conversion reaction [15]:

Co_2_SnO_4_ + 8Li^+^ + 8e^−^ → 2Co + 4Li_2_O + Sn (1)

Sn + 4.4Li^+^ + 4.4e^−^ ↔ Li_4.4_Sn (2)

Sn + 2Li_2_O ↔ SnO_2_ + 4Li^+^ + 4e^−^ (3)

Co + Li_2_O ↔ CoO + 2Li^+^ + 2e^−^ (4)

There was an obvious reduction peak at 0.8 V (vs. Li/Li^+^), which almost disappears in the subsequent cycle, which is attributed to the irreversible decomposition of Co_2_SnO_4_ into Co and Sn, and the formation of solid electrolyte interface film (SEI) on the surface of the active material, thus forming an amorphous Li_2_O matrix, which can be expressed by the reaction. Another reduction peak below 0.2 V (vs. Li/Li^+^) is attributed to the formation of a Li-Sn intermetallic phase. Three anodic peaks were observed at 0.5, 1.2, and 2.0 V (vs. Li/Li^+^), which are attributed to the dealloying process of Li_4.4_Sn, the oxidation of Sn, and the oxidation of Co. In the second and third cycles, based on reversible reaction (4.2), (4.3), (4.4), the three pairs of redox peaks at 0.2/0.5 V (vs. Li/Li^+^), 0.8/1.2 V (vs. Li/Li^+^), and 1.2/2.0 V (vs. Li/Li^+^) correspond to the alloying/dealloying process of Li_4.4_Sn and the redox reaction of Sn/SnO_2_ and Co/CoO, respectively. In addition, the CV curves of peak intensity and integral area of the first cycle and the third cycle almost overlapped, indicating that the co-continuous macroporous structure was the basic framework for the electrochemical reversibility of the redox reaction of Co_2_SnO_4_ materials, and the electrochemical reversibility of Co_2_SnO_4_ materials was gradually established after the initial cycle.

Figure 6b shows the charge-discharge curve of Co_2_SnO_4_-2 electrode at a current density of 200 mA·g^−1^. As observed in CV, the first cycle of the charge-discharge curve was different from that of the subsequent cycle. The electrode showed a large potential platform at about 0.9 V (vs. Li/Li^+^), followed by two tilt potentials below 0.6 V and 0.3 V. The platform at 0.9 V was attributed to the irreversible reduction, amorphization, and decomposition of the original Co_2_SnO_4_ phase and the formation of Li_2_O matrix. The tilt at 0.6 V is attributed to the reaction between the electrode surface and electrolyte, and the tilt at 0.3 V is attributed to the formation of a Li-Sn intermetallic phase [16]. The curve tilted downwards to the cutoff potential of 0.01 V (vs. Li/Li^+^), which indicates the typical characteristic of the voltage trend of Co_2_SnO_4_ electrode. Three platforms of 0.5, 1.4, and 2.0 V were observed during charging, which is attributed to the detachment of Li^+^ and the formation of SnO_2_ and CoO in Li-Sn intermetallic phase [17]. Approximately overlapping charge-discharge curves confirm that Co_2_SnO_4_-2 storage lithium electrode has good reversibility. The first charge-discharge capacity of Co_2_SnO_4_-2 electrode was 1094.3 and 1376.4 mAh·g^−1^, and the initial discharge capacity was much higher than the theoretical capacity of Co_2_SnO_4_ electrode (1105.6 mAh·g^−1^), which was based on the maximum absorption of 12.4 mol Li^+^ per Co_2_SnO_4_. After that, the voltage distribution of charge and discharge in the second and third cycle was different from that in the first cycle, which indicates that the electrochemical reaction process in the first cycle was different from the follow-up cycle of metal oxide materials. In the 10th cycle, the charge-discharge capacity of Co_2_SnO_4_-2 electrode was 1037.1 and 1056.5 mAh·g^−1^, and in the 100th cycle, the charge-discharge capacity of Co_2_SnO_4_-2 electrode was 904.7 and 922.0 mAh·g^−1^. It can be observed that the voltage distribution of Co_2_SnO_4_-2 electrode changed little during the cycle, showing small electrode polarization, capacity attenuation, and good capacity retention. It is worth noting that the uniform and co-continuous macroporous structure and hollow skeleton could effectively alleviate the volume expansion/contraction during the cycle. These results have a great influence on the application value of Co_2_SnO_4_ as electrode material for lithium-ion battery.

As shown in Figure 6c, when the current density increased gradually from 0.1 A·g^−1^ to 0.2, 0.5, 1, 2, and 5 A·g^−1^, the charge-discharge capacity of Co_2_SnO_4_-2 electrode could be maintained at 1281.4/1304.1, 1134.2/1151.9, 1039.9/1052, 949.8/958.3, 861/867.7, and 686/690.1 mAh·g^−1^. Even at the current density of 10 A·g^−1^, the charge-discharge capacity was maintained at 380.6 mAh·g^−1^, showing excellent rate performance.

Figure 6d shows the relationship between the charge capacity, Coulomb efficiency, and the number of cycles of three kinds of Co_2_SnO_4_ electrodes at 200 mA·g^−1^. It was observed that the initial charge-discharge capacity of Co_2_SnO_4_-2 electrode was 1094.3/1376.4 mAh·g^−1^ at low current density, which was slightly lower than that of Co_2_SnO_4_-1 electrode (1146.8/1473.3 mAh·g^−1^) and Co_2_SnO_4_-3 electrode (1132.8/1458.9 mAh·g^−1^). The initial capacity of Co_2_SnO_4_-2 electrode was lower than that of Co_2_SnO_4_-1 electrode and Co_2_SnO_4_-3 electrode, which is attributed to the formation of SEI film on the surface of the skeletons. In the initial cycle, the decrease of cycle stability was due to the partial formation and decomposition of SEI film on the electrode material, which reduced the Coulomb efficiency of the material. After the first cycle, the Coulomb efficiency of the three electrodes was more than 90%. The Co_2_SnO_4_-2 electrode showed good cycle performance, but the capacity of Co_2_SnO_4_-1 and Co_2_SnO_4_-3 electrodes decreased sharply in the first 100 cycles. After 300 cycles, the charge-discharge capacity of Co_2_SnO_4_-2 electrode was 1263.4/1281.7 mAh·g^−1^, which was 115.5% higher than that of the first time. The capacity retention of more than 100% is a common phenomenon in transition metal oxide-based anode materials, which is mainly attributed to the following factors: (1) in the initial charge-discharge process, the contact between active substance and electrolyte is delayed as the number of cycles increases; (2) with the further activation of active substances, the number of active centers increases; (3) the electrolyte penetrates deeply; (4) the extra lithium storage caused by the pseudo-capacitance storage mechanism, which increases the specific capacity of the material. The reversible capacity of Co_2_SnO_4_-1 and Co_2_SnO_4_-3 electrodes after 300 cycles was 434/435.7 and 724.3/729.4 mAh·g^−1^, respectively, and the capacity retention was only 37.8% and 63.9%, respectively. The results show that the co-continuous macroporous structure and hollow skeletons can greatly cushion the volume change of tin-based materials in the process of charge and discharge and significantly improve the cyclic stability of the materials. Compared with the nanoparticle accumulation of “pores” in porous materials and closed porous framework materials, the open porous framework structure had better capacity retention and higher Coulomb efficiency. This can be attributed to the fact that the abundant pore structure maximizes the stress caused by the volume change in the process of lithium removal and intercalation of negative materials, which plays a core role in the excellent cycle performance of Cobalt stannate materials.

The charge-discharge rate was tested with 10 cycles to study the rate performance of the electrodes. As shown in Figure 6e, Co_2_SnO_4_-2 electrode showed better rate charge-discharge performance than Co_2_SnO_4_-1 and Co_2_SnO_4_-3 electrodes. After 5 cycles at current density of 0.1 A·g^−1^, the charge-discharge capacity of Co_2_SnO_4_-2 electrode was from 1298.5/1562.7 mAh·g^−1^ to 1281.4/1304.1 mAh·g^−1^, while that of Co_2_SnO_4_-1 and Co_2_SnO_4_ was from 1117.2/1445.3 to 1081.5/1103 mAh·g^−1^, and from 1171.3/1549.2 to 1131.8/1163.8 mAh·g^−1^, respectively. When the current density increased, the charge-discharge capacity of Co_2_SnO_4_-1 and Co_2_SnO_4_-3 electrode was always lower than that of Co_2_SnO_4_-2. When the current density was reduced to 0.1 A·g^−1^, the specific capacity of Co_2_SnO_4_-2 electrode could rebound to 1090.7 mAh·g^−1^, while that of Co_2_SnO_4_-1 and Co_2_SnO_4_-3 electrodes was only 846.3 mAh·g^−1^ and 925.5 mAh·g^−1^, respectively. In Co_2_SnO_4_ materials, the macroporous structure had excellent magnification properties and effectively suppressed the volume change in the process of charge and discharge; the hollow skeletons provided a conductive channel for ions and electrons, which promoted the electronic conductivity between the electrode material and the electrolyte. It can be seen that the rate performance of Co_2_SnO_4_-2 electrode was better than that of Co_2_SnO_4_-1 electrode and Co_2_SnO_4_-3 electrode.

From the cycle and rate curves, it was found that the cycle test showed a random behavior of capacity, but the rate capability is very smooth. Due to a long-time test (300 cycles or even 500 cycles), any factors such as environmental temperature and humidity changes will cause a fluctuation of the battery capacity, and the cycle curves look somewhat random. It can also be seen that the battery capacity increases after multiple cycles, and the rate curves look smooth, resulting from a short-time cycle test (60 cycles of one week), a relative stable test environment, and the same rate.

In addition, Figure 6f shows the comparison of the reversible capacity and cyclic stability of the synthesized Co_2_SnO_4_ materials with co-continuous macroporous structure and a hollow skeleton with tin-based and cobalt-based oxides published recently [17,18,19,20,21,22,23,24,25,26,27,28]. The prepared Co_2_SnO_4_ has excellent electrochemical performance and a relatively simple preparation method, which further confirms the profound effect of macroporous structure on the electrochemical performance of lithium-ion battery anode materials.

Due to the continuous macroporous structure and hollow skeleton, the Co_2_SnO_4_-2 electrode shows the best long cycle life. Figure 6g shows a long cycle test of all samples in a lithium-ion battery at a high current density of 1 A·g^−1^. After 500 cycles, the specific capacity of Co_2_SnO_4_-2 only decreased from 921.8 to 582.2 mAh·g^−1^, and the capacity retention rate was 63.2%, while the capacity retention rates of Co_2_SnO_4_-1 and Co_2_SnO_4_-3 were 43.1% and 56.0%, respectively. The Coulomb efficiency of Co_2_SnO_4_-2 in the first cycle (78.49%) was higher than that of Co_2_SnO_4_-1 (75.75%) and Co_2_SnO_4_-3 (74.32%), and was significantly higher than that of pure tin (57.2%). It was shown that the prepared Co_2_SnO_4_ materials with continuous macroporous structure and hollow skeleton can be applied to large-scale practical applications.

In addition, the dimensional stability of Co_2_SnO_4_ electrodes after cycling was also studied. The top view and cross section scanning electron microscope images of three kinds of electrodes before and after 50 cycles were compared. The top view (Figure S3a,b,e,f,i,j) proves that Co_2_SnO_4_-2 showed a more flat and uniform surface than Co_2_SnO_4_-1 and Co_2_SnO_4_-3 electrodes, and this surface remained unchanged after electrochemical cycling. There are no obvious signs of cracks, agglomerations, and lithium dendrites, and the structure remained basically unchanged. The texture of small particles distributed on the surface was also related to the formation of SEI and the by-products of electrolyte decomposition, indicating that most of the volume expansion of tin-based materials was buffered in the macroporous structure and hollow skeleton [29]. The cross section (Figure S3c,d,g,h,k,l) shows that Co_2_SnO_4_-2 can maintain the morphology of the electrode. The cross section of the original electrode showed an electrode with a thickness of about 17.92 μm and a uniform particle size distribution. After 50 cycles, the electrode thickness increased slightly to 19.45 μm, and the electrode expansion rate was 8.5%. There is no other obvious structural degradation or weakening of the binding force with the current collector, and the structure remained very good, which was contrary to the situation observed in Co_2_SnO_4_-1 and Co_2_SnO_4_-3. In the other two samples, it was found that the electrode active material was seriously detached from the copper foil, and the thickness of the circulating electrode increased obviously [30]. Itldco be seen that the co-continuous three-dimensional macroporous structure and hollow skeleton had good structural stability, which could effectively restrain the large irreversible volume expansion of anode materials [31]. Therefore, even after a long cycle, the structural integrity of the electrode remained unchanged and there was no obvious performance degradation [32].

**Figure 7:**
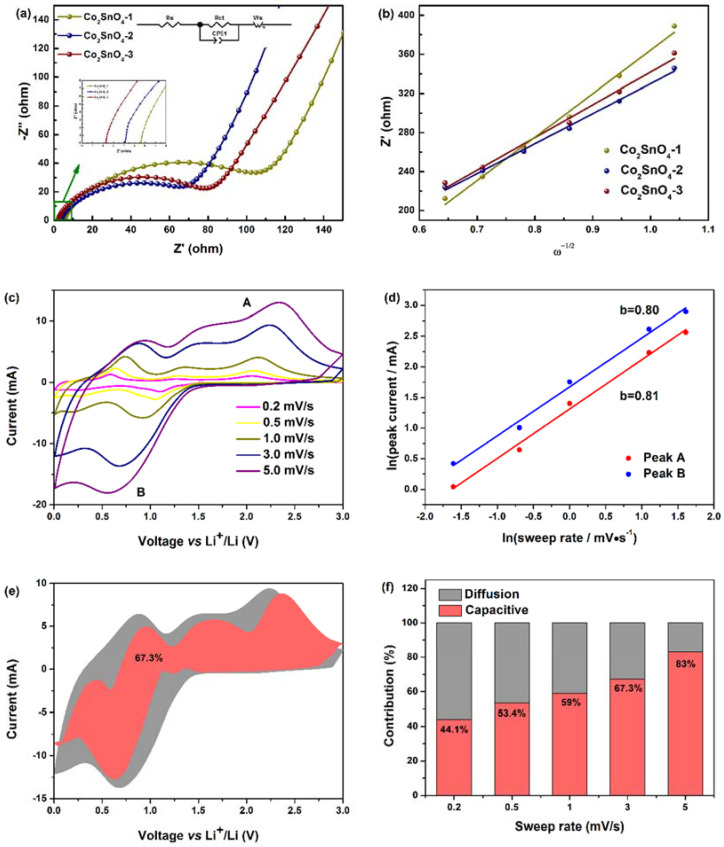
(**a**) Nyquist plots, (**b**) the linear relationship of Z’ and ω^−1/2^ of different anodes, respectively. Inset exhibits the equivalent circuit. (**c**) CV curves at various scan rates. (**d**) Relationship between ln(*i*) vs. ln(*v*). (**e**) Capacitive-contribution at 3.0 mV·s^−1^. (**f**) Contribution ratio of capacitive-controlled capacities at different scan rates for Co_2_SnO_4_-2 anode.

In order to further highlight the structural advantages of the Co_2_SnO_4_ electrodes, the electrochemical impedance spectroscopy (EIS) measurement results of the Co_2_SnO_4_ electrodes are also given in Figure 7 to analyze the electrode kinetic process and battery impedance. The correlation between the morphology and specific surface area of the electrode was analyzed by EIS, which showed the contribution of lithium ion in the electrolyte resistance, charge transfer resistance, and Li^+^ through the solid-state diffusion of the active material. Under the condition of open circuit voltage, the AC impedance spectrum of fresh battery was tested. In Figure 7a, in the frequency range from 100 kHz to 100 mHz, it could be seen that the Nyquist diagram of three Co_2_SnO_4_ electrodes consists of a semicircle in the high/intermediate frequency region and a slope line in the low frequency region. The high frequency region (above 20 kHz) was contributed by ion conduction in electrolyte. Since the Z_img_ part was close to zero, it shows a pure resistance behavior without contribution of the phase constant element. In fact, the semicircle intercept on the axis was the electrolyte solution resistance (R_s_), while the semicircle in the intermediate frequency region (10 kHz–10 Hz) is attributed to the charge transfer resistance (R_ct_) at the electrode/electrolyte interface, and the slope line in the low frequency region (below 10 Hz) corresponded to the diffusion process of lithium ion in the electrode to the active material. The AC impedance spectrum of the experiment was fitted and analyzed by the equivalent circuit model, which was composed of circuit elements of R_s_, R_ct_//CPE1, and W_s_ in series as shown in the illustration [33]. Here, R_s_ is the electrolyte resistance, R_ct_ is the charge transfer resistance, CPE1 is the intercalation capacitance, and W_s_ is the Warburg impedance. The electrolyte resistance (R_s_) of Co_2_SnO_4_-1, Co_2_SnO_4_-2 and Co_2_SnO_4_-3 electrodes was almost the same as that of Co_2_SnO_4_-1, Co_2_SnO_4_-2, and Co_2_SnO_4_-3 electrodes, which was almost the same, but the different values of charge transfer impedance (R_ct_) determined the properties of the active materials, which were 102.30, 65.01, and 77.82 Ω, respectively. The electrochemical impedance of Co_2_SnO_4_-2 electrode was lower than that of Co_2_SnO_4_-1 and Co_2_SnO_4_-3 electrodes, which indicates that the co-continuous macroporous structure and hollow skeleton improved the conductivity and electrochemical performance of the electrode by reducing the charge transfer impedance, which mainly depended on its large specific surface area, thin active material, and continuous wall and pore to provide a shorter and continuous diffusion path for charges/ions. At the same time, it could also inhibit the continuous rupture and formation of the SEI film, make the SEI film more stable, and show a lower charge transfer resistance at the electrode-electrolyte interface, thus maintaining the stability and high charge-discharge cycle of Co_2_SnO_4_ active materials, and improving the Coulomb efficiency [34]. On the basis of the above analysis, all the results also support that the macroporous structure can improve the dynamic properties of the material [35].

The diffusion coefficients of lithium ion on active materials were determined by electrochemical impedance spectroscopy (EIS). The diffusion coefficients were calculated by the following formula:

(5) $$D_{\left({\mathrm{Li}}^{+}\right)}=\frac{R^{2}T^{2}}{2A^{2}n^{4}F^{4}C^{2}\sigma^{2}}$$

In this formula, *n* is the number of electrons per molecule in the oxidation process, *A* is the surface area of the active substance, *D* is the diffusion coefficient of lithium ion, *R* is the gas constant, *T* is the absolute temperature, *F* is the Faraday constant, *C* is the lithium ion concentration, and *σ* is the Warburg impedance coefficient. The value of *σ* is calculated based on the slope of the linear curve (0.38–0.14 Hz) between the real part of the impedance (*Z*’) and the inverse square root of the frequency (*ω*^−1^), as shown in Figure 7b. The diffusion of Li^+^ in active materials can be calculated from the data obtained in the low frequency region at a phase angle of 45° to the real axis. The Warburg impedance coefficient (*σ*) is related to the Warburg factor, which is given by the following formula:

(6) $$Z_{re}=R_{ct}+R_{s}+\sigma \omega^{-1/2}$$

As shown in Table S1, the calculated values of Li^+^ diffusion coefficients of Co_2_SnO_4_-1, Co_2_SnO_4_-2, and Co_2_SnO_4_-3 were 7.94 × 10^−19^, 1.67 × 10^−18^, and 1.40 × 10^−18^ cm^2^·s^−1^, respectively. Co_2_SnO_4_-1 had the lowest diffusion coefficient, followed by Co_2_SnO_4_-3, and Co_2_SnO_4_-2, which had the highest diffusion coefficient, which indicates that the electrode kinetics of lithium ion was the strongest. This is consistent with the structural and electrochemical characterization. From this result, it can be concluded that the macroporous structure is one of the important strategies to enhance the Li^+^ diffusion by increasing the electronic conductivity of the material.

In order to further study the kinetic factors affecting electrochemical performance, the cyclic voltammetry curve of Co_2_SnO_4_-2 was measured at a series of scanning rates of 0.2, 0.5, 1, 3, and 5 mV·s^−1^. As shown in Figure 7c, when the scanning speed increased, the characteristic redox peak could still be observed, and the redox peak current increased, indicating the fast reaction kinetics, high reversibility, and high-rate performance of Li^+^ storage. In the CV test, the electrochemical reaction could be divided into diffusion control and pseudo-capacitance control processes, according to the following equations:

*i* = a*v*^b^ (7)

ln(*i*) = bln(*v*) + ln(a) (8)

where *i* is the peak current (mA), *v* is the scanning rate (mV·s^−1^), a and b are adjustable parameters, and the lithium storage behavior of Co_2_SnO_4_-2 electrode could be determined by b value (b is calculated by the slope of ln(*i*) vs. ln(*v*)). In general, b = 0.5 means that the capacity is controlled by diffusion behavior, and b = 1 means that the electrochemical reaction is controlled by the pseudo-capacitive process. As shown in Figure 7d, there was a good linear relationship between ln(*i*) and ln(*v*) in the range of 0.2~5 mV·s^−1^. The b values of oxidation peak and reduction peak of Co_2_SnO_4_-2 were 0.81 and 0.80, respectively, indicating that Co_2_SnO_4_-2 electrode had mixed lithium storage behavior: diffusion-controlled alloying reaction and pseudo-capacitance process jointly controlled the electrochemical reaction. In addition, Co_2_SnO_4_-2 electrode achieved high rate capacity at high current density, which may be related to the pseudo-capacitance control mechanism of lithium storage. The pseudo-capacitance contribution can be determined by the following formula [36]:

*i*(*v*) = k_1_*v* + k_2_*v*^1/2^ (9)

where *i*(*v*) is the total current response, k_1_*v* and k_2_*v*^1/2^ represent the pseudo-capacitance control and diffusion control process at a given voltage *v*. The values of k_1_ and k_2_ are calculated after drawing the relationship between *v*^1/2^ and *i*/*v*^1/2^. Figure 7e shows the contribution rate of pseudo-capacitive control charge and diffusion control charge at different scanning rates. At the scanning rates of 0.2, 0.5, 1, 3, and 5 mV·s^−1^, the contribution of pseudo-capacitance accounted for 44.1%, 53.4%, 59%, 67.3%, and 83% of the total capacity of the Co_2_SnO_4_-2 electrode, respectively. The results show that at lower scanning rate, the capacity of Co_2_SnO_4_-2 electrode mainly came from the contribution of diffusion dominant process, which is mainly due to the rapid diffusion of lithium ion (porous structure). At a higher scanning rate, the pseudo-capacitance behavior played a leading role in the storage/release of lithium ions, and the pseudo-capacitance storage mechanism had fast charge-discharge characteristics [37]. This behavior can be attributed to the fact that the large specific surface area of Co_2_SnO_4_ enabled lithium ions to insert and detach quickly, and also enhanced the relationship between the electrolyte and the active material [38]. On this basis, the pseudo-capacitance behavior of Co_2_SnO_4_ electrode was improved. The dynamic evaluation further explains the reason for the high-rate performance under high current [39].

## 3. Conclusions

The double transition metal hydroxide material with co-continuous macroporous structure and hollow skeletons was successfully synthesized by sol-gel method combined with phase separation, and Co_2_SnO_4_ was obtained after heat treatment in air atmosphere at 550 °C. The resultant macroporous Co_2_SnO_4_ with hollow skeletons showed intriguing attributes of high surface area, appropriate pore size, ideal electrolyte wettability, and excellent conductivity. The resultant macroporous Co_2_SnO_4_ had high cyclic stability. After 300 cycles, the initial capacity of macroporous Co_2_SnO_4_ was even maintained at about 115.5%. At the high current density (1 A·g^−1^) test, the macroporous Co_2_SnO_4_ showed a high specific capacity of 921.8 mAh·g^−1^, and only decreased by 36.8% after 500 cycles. The macroporous Co_2_SnO_4_ with hollow skeletons could not only effectively buffer the volume change of tin-based materials without introducing excess aggregation and loss, but also promoted the diffusion of Li^+^, increased the specific capacity of materials to some extent, and thus had stronger energy storage performance. The simple preparation route of sol-gel method combined with phase separation provided the possibility for large-scale commercial production.

## 4. Materials and Methods

### 4.1. Synthesis

Cobalt (II) chloride anhydrous (CoCl_2_, Sigma-Aldrich, Shanghai, China, ≥99.7%), stannous chloride dihydrate (SnCl_2_·2H_2_O, Sigma-Aldrich, Shanghai, China, 98%), glycerol (C_3_H_8_O_3_, Sinopharm Chemical Reagent Co., Ltd., Shanghai, China, AR), poly (acrylic acid) (PAA, Sigma-Aldrich, Shanghai, China, 35 wt% in water, average molecular weight of 100,000), propylene oxide (PO, Sinopharm Chemical Reagent Co., Ltd., Shanghai, China, AR), and ethanol absolute (C_2_H_6_O, Sinopharm Chemical Reagent Co., Ltd., Shanghai, China, AR) were used as analytical grade reagents without further purification.

Crystalline Co_2_SnO_4_ was synthesized by a sol-gel method accompanied by phase separation. Firstly, 0.7790 mg of cobalt (II) chloride anhydrous (CoCl_2_) and 0.6768 mg of stannous chloride dihydrate (SnCl_2_·2H_2_O) were dissolved in 1.82 mL of ethanol absolute and 2 mL of glycerol containing poly (acrylic acid) (PAA). This solution was stirred at room temperature for 30 min. Then, 1.88 mL propylene oxide (PO) was added into the zinc and tin precursor solution slowly under stirring conditions to form gel. The sol-gel transition was intense. The gel was aged at 60 °C for 24 h and immersed in ethanol absolute for 24 h at 60 °C three times. The crystalline Co_2_SnO_4_ was obtained after drying at 60 °C for 48 h and heat-treating at 550 °C in air for 2 h.

Three kinds of Co_2_Sn(OH)_6_-1, Co_2_Sn(OH)_6_-2, and Co_2_Sn(OH)_6_-3 xerogels were prepared by controlling the amount of PAA at 0.71, 1.43, and 2.14 g, and Co_2_SnO_4_-1, Co_2_SnO_4_-2, and Co_2_SnO_4_-3 materials were obtained after heat treatment.

### 4.2. Characterizations

The microstructure and morphology of the as-obtained materials were obtained through field emission scanning electron microscopy (FESEM, SU-8010, Hitachi, Tokyo, Japan), transmission electron microscopy (TEM, JEM-2100, JEOL, Tokyo, Japan) coupled with energy-dispersive X-ray spectroscopy (EDS). X-ray diffraction (XRD) was conducted on a Bruker D8 ADVANCE XRD in the 2*θ* range from 10° to 90°. X-ray photoelectron spectroscopy (XPS) measurements were executed with mono-chromated Al K alpha by using an ESCALAB 250Xl (ThermoFisher Scientific, Waltham, MA, USA). The Brunauer–Emmett–Teller (BET, ASAP2460, Micromeritics Instruments Corporation, Norcross, GA, USA) method result was achieved by nitrogen adsorption- desorption experimental points at a relative pressure (*P*/*P*_0_ = 0.05–0.25), and the macropore size distribution was recorded using a mercury injection apparatus (AutoPore IV 9510, Micromeritics Instruments Corporation, Norcross, GA, USA). Thermo-gravimetry (TG) and differential scanning calorimetry (DSC) measurements were performed at TG–DSC (STA449F3 Jupiter, NETZSCH-Gerätebau GmbH, Selb, Germany) at a heating rate of 10 °C min^−1^ in air.

### 4.3. Electrochemical Measurements

Conventional electrodes were fabricated with CMC as the binder and Super P as the conductive additive. The weight ratios of the active materials, Super P, and CMC were 7:2:1, and these constituents were mixed in deionized water. The resulting slurry was cast on current collectors (Cu foil) and dried at 60 °C for 6–8 h. On average, the loading mass was 1.2 mg·cm^−2^. The 2032-type coin cells were assembled in an argon-filled glove box (moisture level < 1.0 ppm) (Super(1220/750/900), MIKROUNA, Shanghai, China). Half cells were assembled using lithium metal as the counter electrode and Celgard 2350 as the separator. A 1.0 M LiPF_6_ solution in a 1:1:1 mixture of DMC, EMC, and FEC was employed as the electrolyte. Galvanostatic charge-discharge measurements were carried out using a battery cell test system (CT2001A, LAND, Wuhan, Chian). The C-rate capability was estimated by varying the discharge current density from 0.1 to 10 A·g^−1^. Cyclic voltammetry (CV) and electrochemical impedance spectroscopy (EIS) measurements were carried out using an electrochemical workstation (CHI760E, Chenhua, Shanghai, China). Half cells were cycled in the voltage range of 0.01–3.0 V.

## Figures and Tables

**Table 1:** The surface area and pore size distribution of Co_2_SnO_4_-1, Co_2_SnO_4_-2, and Co_2_SnO_4_-3, respectively.

Sample	SSA (m^2^·g^−1^)				Vadsorption (cm^3^·g^−1^)	APD (nm)
	Stotal	Smicro	Sext	Ratio		
Co_2_SnO_4_-1	18.04	2.79	15.25	85%	0.11	23.41
Co_2_SnO_4_-2	22.31	2.03	20.28	91%	0.13	31.74
Co_2_SnO_4_-3	16.55	2.39	14.16	86%	0.10	28.47

## Data Availability

The data presented in this study are available on request from the corresponding author.

## Supplementary Materials

The following supporting information can be downloaded at: https://www.mdpi.com/article/10.3390/gels8050257/s1, Figure S1: Full XPS spectra for Co_2_SnO_4_-1, Co_2_SnO_4_-2 and Co_2_SnO_4_-3; Figure S2: TG-DSC curves of (a) Co_2_Sn(OH)_6_-1, (b) Co_2_Sn(OH)_6_-2 and (c) Co_2_Sn(OH)_6_-3 from room temperature to 750 °C in air atmosphere; Figure S3: The ex situ SEM images of the (a and b) Co_2_SnO_4_-1, (e and f) Co_2_SnO_4_-2 and (i and j) Co_2_SnO_4_-3 anodes surface before and after 50 cycles, respectively; The cross-sectional SEM images of the (c and d) Co_2_SnO_4_-1, (g and h) Co_2_SnO_4_-2 and (k and l) Co_2_SnO_4_-3 anodes surface before and after 50 cycles, respectively; Table S1: The R_s_, R_ct_ and lithium ion diffusion coefficients D(Li^+^) of different anodes.
