# Supplementary material for: Construction of Macroporous Co2SnO4 with Hollow Skeletons as Anodes for Lithium-Ion Batteries
Source: Gels. 2022 Apr 21;8(5):257. doi: 10.3390/gels8050257 (PMC9140520; doi:10.3390/gels8050257)
Supplement: Supplementary file 1 [file gels-08-00257-s001.zip › gels-1676845-supplementary.pdf]

gels

MDPI

Article

Construction of Macroporous Co_2SnO_4 with Hollow Skeletons as Anodes for Lithium-Ion Batteries

Jintian Wang [1], Junzhang Wang [1], Xingzhong Guo [1,2,*] and Hui Yang [1,2]

Supporting Information

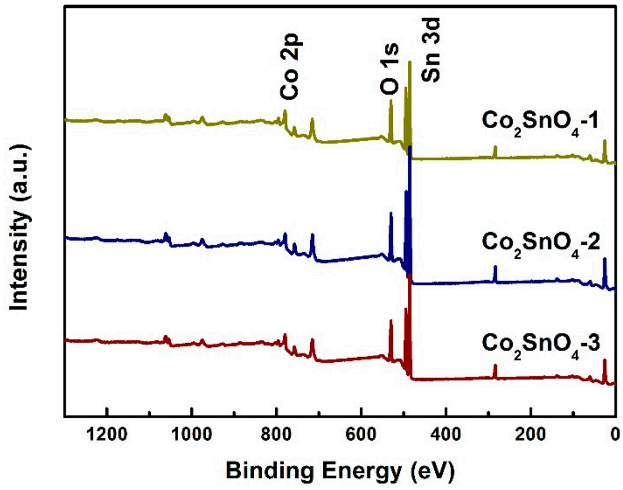

Figure S1. Full XPS spectra for Co_2SnO_4-1, Co_2SnO_4-2 and Co_2SnO_4-3.

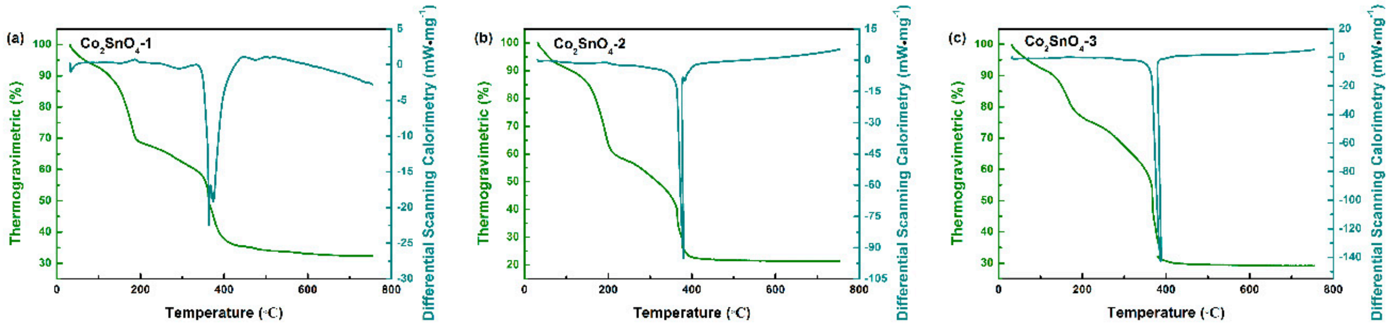

Figure S2. TG-DSC curves of (**a**) $Co_2Sn(OH)_6$-1, (**b**) $Co_2Sn(OH)_6$-2 and (**c**) $Co_2Sn(OH)_6$-3 from room temperature to 750 °C in air atmosphere.

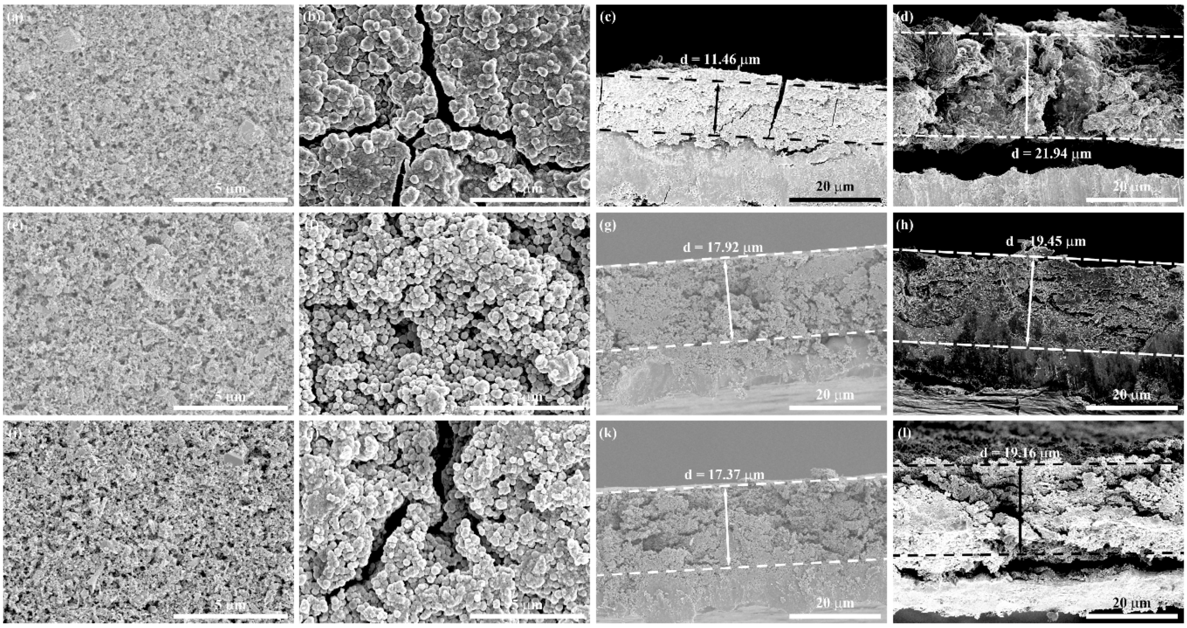

Figure S3. The *ex situ* SEM images of the (**a** and **b**) Co_2SnO_4-1, (**e** and **f**) Co_2SnO_4-2 and (**i** and **j**) Co_2SnO_4-3 anodes surface before and after 50 cycles, respectively; The cross-sectional SEM images of the (**c** and **d**) Co_2SnO_4-1, (**g** and **h**) Co_2SnO_4-2 and (**k** and **l**) Co_2SnO_4-3 anodes surface before and after 50 cycles, respectively.

Table S1. The R_s, R_{ct} and lithium ion diffusion coefficients $D(Li^+)$ of different anodes.

Electrodes	Rs（Ω）	Rct（Ω）	Slope（$Z'\sim\omega^{-1/2}$）	D（Li^+）（$cm^2\cdot s^{-1}$）
Co_2SnO_4-1	5.59	102.30	444.74	7.94×10^{-19}
Co_2SnO_4-2	4.09	65.01	306.90	1.67×10^{-18}
Co_2SnO_4-3	2.28	77.82	334.73	1.40×10^{-18}
